# Real-World Analysis of Olanzapine and Functional Outcomes in Outpatient Cancer Rehabilitation

**DOI:** 10.3390/cancers18182943

**Published:** 2026-09-11

**Authors:** Ioanna Karras, Kayla Beck, Addison Barber, Kathryn Abplanalp, Ishan Roy

**Affiliations:** 1 Shirley Ryan AbilityLab, Chicago, IL 60611, USA; ikarras@arizona.edu (I.K.); kbeck@sralab.org (K.B.); abarber@sralab.org (A.B.); kabplanalp@sralab.org (K.A.); 2 Edward Hines Jr. Veterans Administration Hospital, Maywood, IL 60141, USA; 3 Department of Physical Medicine and Rehabilitation, Northwestern University, Chicago, IL 60208, USA; 4 Robert H. Lurie Comprehensive Cancer Center, Northwestern University, Chicago, IL 60611, USA

**Keywords:** cancer, cachexia, appetite, olanzapine, rehabilitation

## Abstract

**Background/Objectives**: Cachexia is a multifactorial muscle wasting syndrome driven by inflammation, causing increased catabolism and decreased food intake. Using appetite stimulation as part of multidisciplinary rehabilitation for cachexia is an emerging strategy. Olanzapine was originally developed as an atypical antipsychotic and has since emerged in rehabilitative strategies in patients with brain injury and chemotherapy-induced nausea. In 2023, the American Society of Clinical Oncology (ASCO) issued a rapid recommendation for the use of low-dose (2.5–5 mg) olanzapine to improve appetite and weight in adults with advanced cancer cachexia, based on novel evidence of increased appetite and weight gain. **Methods**: We retrospectively evaluated the impact of olanzapine on a real-world cohort of 45 patients with appetite concerns undergoing cancer-related rehabilitation. Patients were retrospectively evaluated for changes in body composition metrics and physical therapy (PT) related outcomes. **Results**: While treatment groups were well matched at baseline, there were no statistical differences between group outcomes, including weight gain, contrary to findings in other populations. However, fatigue was a significant predictor of PT goal completion across the cohort (*p* = 0.017), highlighting the need for future studies to investigate this relationship more deeply. **Conclusions**: These results indicate that the effects of olanzapine may vary between different clinical populations and suggest that further investigation into adjuvant therapies remains critical for optimizing care for cancer patients with anorexia.

## Simple Summary

Cancer cachexia is a muscle wasting condition that affects 50–80% of all cancer patients. Patients with cachexia have poorer clinical outcomes, lower quality of life, greater disability, and decreased cancer treatment tolerance. Reduced appetite is a hallmark of cachexia, and low-dose olanzapine has recently been recommended to improve appetite and promote weight gain in patients with advanced cancer cachexia. However, there are no studies evaluating the effect of olanzapine on patients with appetite disturbances who are undergoing active physical rehabilitation. This pilot study aimed to bridge that gap by comparing real-world physical therapy outcomes of cancer patients with appetite concerns taking olanzapine to those who were not. There were no significant differences in physical therapy outcomes, body weight, or body composition between the two groups after rehabilitation. However, baseline fatigue was a significant predictor of physical therapy success. These findings suggest that the efficacy of olanzapine may vary among cancer cachexia populations.

## 1. Introduction

Cancer cachexia is a complex, multisystem condition that affects 50–80% of all cancer patients [1]. Cachexia is characterized by the loss of skeletal muscle, physical function, and appetite, which cannot be fully addressed by current nutritional support strategies [2,3]. A major driver of cachexia is systemic inflammation, which further exacerbates functional impairments, decreases quality of life, and reduces tolerance to cancer treatment [4,5,6]. There is increasing evidence that multidisciplinary approaches, involving rehabilitation and nutritional support, can best preserve physical function in this population [7].

The heterogeneous phenotype of the condition, however, has made it challenging to develop a clear treatment protocol. Managing cancer-related anorexia and muscle loss remains at the center of this challenge. In 2023, the American Society of Clinical Oncology (ASCO) issued a rapid recommendation for the use of low-dose olanzapine, 2.5–5 mg once daily, in adults with advanced cancer to improve appetite and body weight [8]. Olanzapine is an atypical antipsychotic originally developed for the treatment of schizophrenia [9]. Olanzapine has an affinity for several dopamine, adrenergic, muscarinic, serotonin, and histamine receptors, with the latter two most closely linked to its orexigenic properties [10,11]. Over 90% of patients taking olanzapine gain weight, and it was this observation that initially drew interest into utilizing olanzapine as an appetite stimulant in cancer cachexia [12,13]. Additionally, growing evidence of its efficacy in improving patient outcomes in other rehabilitative settings made it a convincing potential therapeutic in oncology [14].

While ASCO had previously reviewed evidence for prescribing olanzapine for the treatment of advanced cancer cachexia in 2020, they did not find sufficient supporting data at the time [15]. However, a randomized, placebo-controlled study published in May 2023 found that treatment with low-dose olanzapine resulted in improved appetite and weight gain in patients with advanced cancer who were beginning chemotherapy [16]. Another recent trial in a different population also observed overall initial decreases in anorexia, though this effect was not sustained throughout the interventional period compared to the control group. While there was an increase in body weight with olanzapine, hand grip strength scores decreased [17]. A meta-analysis of several cancer cachexia studies investigating olanzapine treatment found that while body weight increased in the interventional arm overall, this effect did not achieve statistical significance [18]. Such results underscore the need for comprehensive assessment of body composition, functional outcomes, physical therapy completion, cancer type and stage, and other relevant impairments when developing therapeutic strategies.

Evidence is particularly lacking when considering olanzapine in the context of active physical therapy (PT). It is increasingly clear that exercise and PT can improve cancer outcomes and maintain functional capacity in individuals with cancer cachexia and appetite disturbances [19,20]. These forms of physical activity likely combat cachexia by decreasing systemic inflammation while increasing muscle function and cellular metabolism [21]. The specific modalities of exercise that are most effective remain largely unknown and will likely require personalization in tandem with nutritional support [22]. Careful monitoring is required to ensure that patients do not enter a negative energy balance due to the intensity of the physical activity [23]. Several additional challenges exist in the rehabilitation of patients with cancer cachexia. One real-world study evaluating cancer cachexia care in Germany found that half of patients did not have clearly defined goals in nutritional and physical therapy, which decreases therapy efficacy [24]. Furthermore, the recovery of physical function is often hindered by other impairments that exercise and appetite stimulation alone cannot address [25,26]. Multimodal rehabilitation emphasizes active collaboration between the spectrum of specialties involved in cancer cachexia treatment and has been demonstrated to improve quality of life in patients with cancer cachexia [27,28]. Such integration will become increasingly necessary as treatment options for cachexia continue to broaden. Better understanding these intersections in different patient populations will be particularly crucial in developing individualized treatment plans. Relevantly, while olanzapine has been shown to increase body weight in certain cancer populations, there is no literature investigating it in the context of rehabilitation.

The purpose of this study was to evaluate the impact of olanzapine on body composition and PT completion in cancer patients with appetite disruption undergoing clinical rehabilitation. As patients referred to this clinic have a variety of physical impairments that have distinct associated outcome measures that are not always captured via standard functional assessment batteries, we assessed physical function by measuring the number of PT goals that were accomplished for each patient. We hypothesize that while olanzapine would improve overall body weight, its influence on physical outcomes would be mediated by baseline skeletal muscle mass. We further examine the effect of common comorbidities and impairments on PT outcomes to consider the multifactorial nature of functional recovery.

## 2. Materials and Methods

### 2.1. Data Collection and Processing

A retrospective chart review (Northwestern University Institutional Review Board, STU00219423) was conducted on 405 patients presenting to clinic for cancer-related rehabilitation between September 2022 and July 2025. Inclusion criteria included patient-reported appetite disturbances during medical evaluation with a clinic physician. Additional criteria for inclusion were the referral to and completion of a course of physical therapy after the physician encounter. In total, 72 patients had documented appetite issues and were referred for physical therapy (PT) evaluation. Of these, 27 patients were excluded as their data was inaccessible due to completing their PT externally or not at all, resulting in missing data. A final cohort of 45 patients with complete data was established.

The patients were then stratified into two groups: those receiving olanzapine (OLZ+, *n* = 20) and those not receiving olanzapine (OLZ−, *n* = 25). As the July 2023 ASCO Rapid Recommendation was released within the study period, the cohort included 9 exclusively OLZ− patients who initiated rehabilitation between September 2022 and July 2023. After the recommendation, the sample included a combination of 20 OLZ+ patients and an additional 16 OLZ− patients. Of these 16 patients, the majority did not receive olanzapine as they declined the medication when it was offered at evaluation. Apart from 1 patient who disclosed having stopped taking olanzapine shortly after beginning PT, all other OLZ+ patients are presumed to have adhered to the treatment throughout PT. Additionally, 10 patients (6 OLZ+ and 4 OLZ−) were concurrently taking alternate appetite stimulants, including mirtazapine, megestrol acetate, or cannabinoids.

Clinical and research staff collected patient demographics, cancer status and treatment history, impairments, perceived independence with activities of daily living (ADLs) and instrumental activities of daily living (IADLs) at the first clinical encounter. Body composition was assessed using the BWA 2.0 Body Composition Analyzer (InBody, Cerritos, CA, USA). Individual clamps were placed on each limb while patients remained in a seated position while the BWA 2.0 conducted two scans. Phase values from each frequency were confirmed to be non-conflicting. Using patient height and weight, collected on the same day, their SMI was automatically calculated by the included software. Nine patients (3 OLZ+ and 6 OLZ−) were evaluated at a clinic location with no access to the BIA 2.0 and had no body composition data. Eleven additional patients (5 OLZ+ and 6 OLZ−) did not have body composition data at completion of PT, as they did not return to clinic for a follow-up visit.

All patients then underwent a PT evaluation with a physical therapist, where PT goals were developed and collected and later organized by type by the researchers. Since physical therapists had access to patient electronic health records, they were not blinded to olanzapine status. Manual muscle testing (MMT) scores were also evaluated by the physical therapist, when appropriate, for Hip Abduction (7 OLZ+ and 8 OLZ−), Hip Extension (8 OLZ+ and 5 OLZ−), and Hip Flexion (13 OLZ+ and 14 OLZ−). Changes in body weight (BW), skeletal muscle index (SMI) via the BIA 2.0, and prognostic nutritional index (PNI) were used to track physical changes. Both the Fearon criteria, defined as greater than 5% unintentional weight loss within the past six months, and the weight loss grading scale (WLGS), which incorporates both percent of weight loss and BMI to grade cachexia severity from zero to four, were used to evaluate cachexia status [3,29]. At the final PT session (last PT), goals were re-assessed by the physical therapist for completion. The number, proportion, and type of PT goals completed were used to assess functional outcomes.

### 2.2. Statistical Analysis

Statistical analyses were conducted using IBM SPSS Statistics 31 and GraphPad Prism 10. The baseline characteristics, impairments, and cancer data of both treatment groups were compared to ensure there were no significant differences between group distributions. Sensitivity analyses adjusting for breast cancer status were conducted, as this was significantly different between the treatment groups. Continuous variables were compared using independent samples *t*-tests, and categorical variables were compared using Fisher’s exact tests. Next, treatment group outcomes were compared to each other and to their respective baseline measurements. Mixed effect analyses were used when missing values were present, and two-way ANOVA when a complete data set was available.

Models were then developed to compare assigned PT goals to those completed while controlling for individual covariates. Covariates were chosen based on their known clinical relevance to cancer cachexia rehabilitation outcomes, and the resulting estimated marginal means were compared. Variations in group size across models reflect the available data for those specific metrics. To preserve statistical power given the small sample size, separate binomial generalized linear models were used per covariate. A Pearson chi-square dispersion scale parameter was applied to adjust standard errors and account for the overdispersion in the cohort.

## 3. Results

### 3.1. Baseline Patient Characteristics and Group Comparison

**Figure 1:**
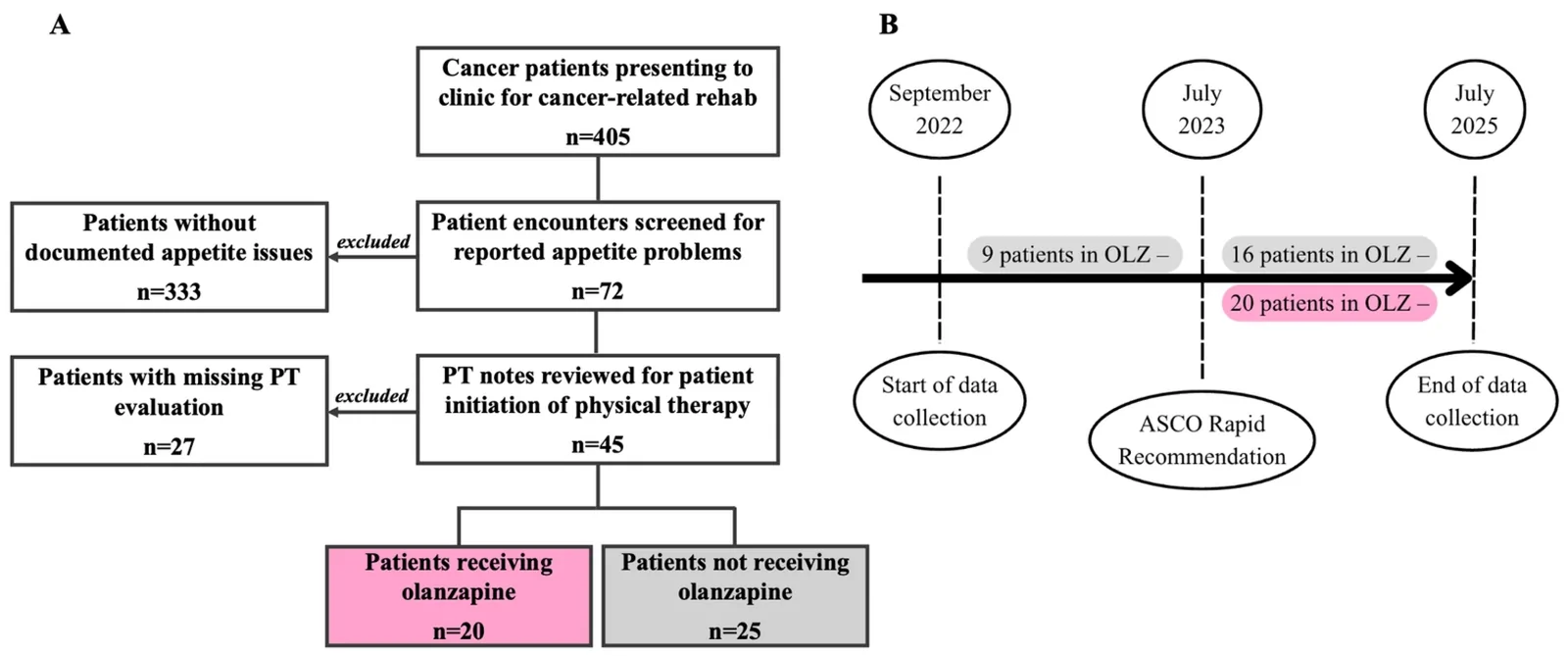
Observational-Study Participant Flow Diagram and Study Timeline. (**A**) Consort diagram illustrating the inclusion and exclusion criteria of the chart review process. (**B**) Study timeline visualizing the distribution of patients within treatment groups prior to the 2023 American Society of Clinical Oncology (ASCO) rapid recommendation and after.

This retrospective study had a final sample of 45 patients, comprising 20 OLZ+ patients and 25 OLZ− patients (Figure 1). There were no statistically significant differences in baseline demographics, cancer stage, clinical impairments, anthropometrics, nor cachexia status as measured by the Fearon criteria and the WLGS (Table 1). There were more patients in both groups who met both the Fearon and WLGS criteria for cachexia than those who did not. However, there was increased prevalence of breast cancer in the OLZ− group (*p* = 0.027).

### 3.2. Clinical Markers of Cachexia

**Figure 2:**
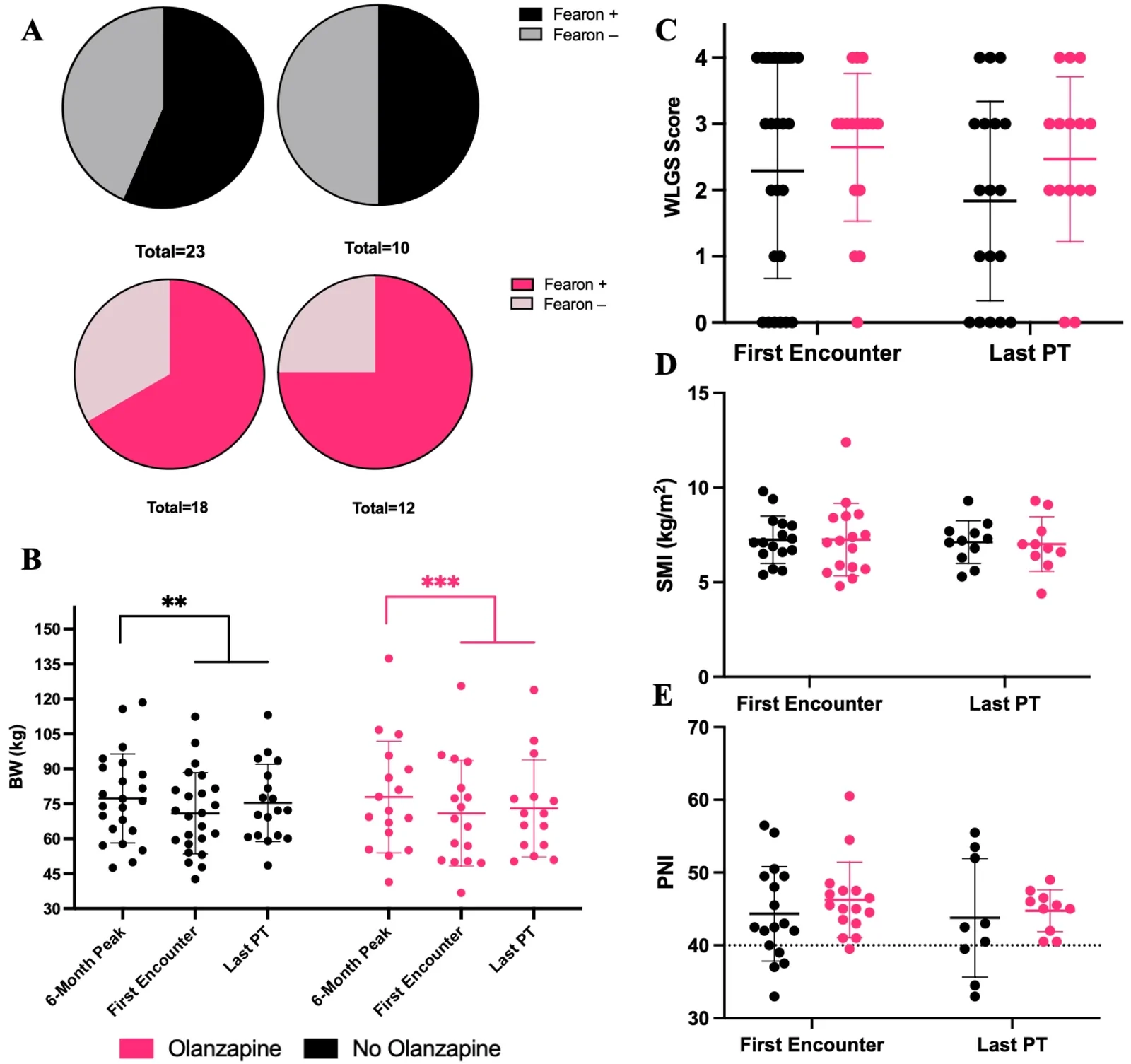
Comparison of cohort differences in musculoskeletal and biomarker baseline and outcome measurements. (**A**) Visualization of Fearon criteria status between groups and after rehabilitation. The upper pie charts represent OLZ− patients, while the bottom pie charts represent OLZ+ patients. The left pie charts represent Feraon criteria status at first encounter and the right pie charts show data from the last physical therapy (PT) session. (**B**–**E**) Comparisons of (**B**) body weight (BW) (OLZ-: 6-Month Peak, *n* = 23, First Encounter, *n* = 24, Last PT, *n* = 18; OLZ+: 6-Month Peak, *n* = 17, First Encounter, *n* = 17, Last PT, *n* = 15), (**C**) weight loss grading scale (WLGS) score (OLZ-: First Encounter, *n* = 24, Last PT, *n* = 18; OLZ+: First Encounter, *n* = 17, Last PT, *n* = 15), (**D**) skeletal muscle index (SMI) (OLZ−: First Encounter, *n* = 16, Last PT, *n* = 11; OLZ+: First Encounter, *n* = 15, Last PT, *n* = 10), and (**E**) prognostic nutritional index (PNI) between first encounter and last PT session (OLZ−: First Encounter, *n* = 17, Last PT, *n* = 9; OLZ+: First Encounter, *n* = 16, Last PT, *n* = 10). (**B**) includes each patient’s maximum recorded weight in the 6 months prior to initiating physical therapy (6-Month Peak) (** *p* < 0.01, *** *p* < 0.001). The dashed line in (**E**) indicates the clinical threshold for PNI. Across all panels, pink indicates the OLZ+ group, and black indicates the OLZ− group. All lines denote mean and SD error bars.

Following the baseline assessment, as part of routine care in the clinic, changes were tracked in primary cachexia markers from the first encounter through the completion of rehabilitation to evaluate the impact of olanzapine. The average time to PT evaluation from beginning olanzapine was 22 days, with a standard deviation of 30 days. The range was −34 to 95 days, as 3 patients began olanzapine treatment after initiating PT, but before PT completion. There were no statistically significant differences in Fearon status or WLGS score between groups or with rehabilitation (Figure 2A). Significant weight loss was present in the 6-month period prior to the first encounter in clinic in both groups, with mixed effect analysis revealing a mean decrease of 6.98 kg in the OLZ+ group (*p* = 0.0002) and a mean decrease of 5.87 kg in the OLZ− group (*p* = 0.0007). (Figure 2B). Overall, clinical markers of cachexia status remained unchanged after completion of PT. In the overall 45-patient cohort, there were no significant changes in the overall BW, SMI, or PNI after rehabilitation (*p* ≥ 0.45). Mixed effect analyses revealed no statistically significant differences in BW, WLGS, SMI, or PNI with olanzapine (*p* ≥ 0.28), over rehabilitation (*p* ≥ 0.19), or the interaction term (*p* ≥ 0.68). (Figure 2B–E). .

### 3.3. Functional Outcomes with Olanzapine

**Figure 3:**
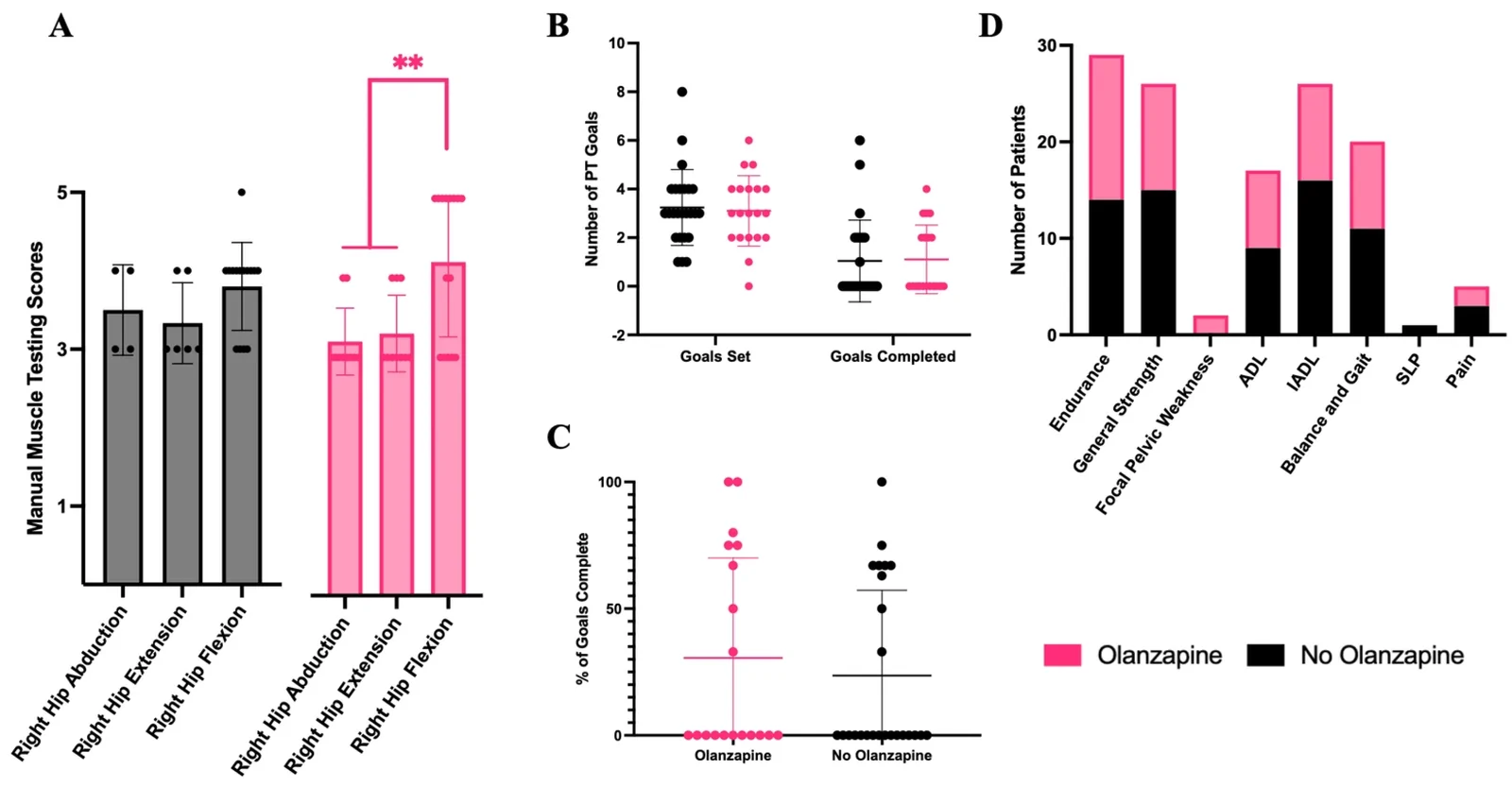
Impairment status and rehabilitation outcomes with and without olanzapine. (**A**) Manual Muscle Testing (MMT) scores for hip abduction, extension, and flexion. 2-way ANOVA was used to compare the two groups, 3 muscle types, and any interaction effects (** *p* < 0.01). (**B**) Number of PT goals set compared to number of goals completed. (**C**) Proportion (%) of total goals completed stratified by treatment group. (**D**) Distribution of the drivers of goals during PT. Across all panels, pink indicates the OLZ+ group, and black indicates the OLZ− group. All lines denote mean and SD error bars.

We first examined baseline functional status as it may have impacted cachexia clinical markers. MMT scores were analyzed to determine if there were differences between groups in muscle strength prior to PT initiation. Right leg values were used for all patients to maintain consistency. While there were no statistically significant differences between groups (*p* ≥ 0.0978), OLZ+ patients had significantly lower values in hip abduction and extension, compared to hip flexion (*p* ≤ 0.004). OLZ− patients did not show any significant differences across muscle groups (*p* ≥ 0.31) (Figure 3A).

Functional outcomes after rehabilitation were then evaluated. There was no difference in the number of goals set at the first PT session between the OLZ+ (mean = 3.2 ± 1.6) and OLZ− (mean = 3.1 ± 1.4) (*p* = 0.8). At completion of PT, both OLZ+ (mean = 1.1 ± 1.4) and OLZ− (mean = 1.0 ± 1.7) had completed the same average number of goals (*p* = 0.96) (Figure 3B). The OLZ+ group had completed a mean of 23.5% of PT goals, compared to 30% of the OLZ− group, though this was not a significantly different proportion (*p* = 0.54). There was also no statistically significant difference in variance (*p* = 0.46) (Figure 3C). To determine if there were fundamental differences in the types of goals and exercises undertaken by the two groups, the drivers of goals that determined specific PT exercises were assessed. However, there were no statistically significant differences in drivers between groups (*p* = 0.14) (Figure 3D).

### 3.4. Analysis of Covariates

Baseline covariates were assessed as potential predictors to the proportion of completed PT goals. Individually generated beta-binomial models were used to assess covariates while accounting for overdispersion of the sample. The results revealed no significant effect for demographics such as age and sex (Table 2). Common physical impairments, including ataxia, neuropathy, and pelvic weakness, were not significant predictors, and neither were SMI, Fearon status, or the ability to complete ADLs (*p* > 0.05). However, a patient receiving a WLGS score ≥ 3 was significantly associated with decreased goal completion (*p* = 0.022, Exp (B) = 0.255). Finally, fatigue was a statistically significant covariate, with fatigue being associated with 72.9% lower odds of completing PT goals (*p* = 0.017, Exp (B) = 0.271).

We then reran our analysis, excluding the 3 patients who had begun olanzapine prior to initiating PT. Significance and effect direction persisted for WLGS ≥ 3 (*p* = 0.012, Exp (B) = 0.199) and fatigue (*p* = 0.038, Exp (B) = 0.270). Another analysis was conducted, this time excluding the 10 patients who were on alternative appetite stimulants. Once again, fatigue remained significantly associated with decreased odds of completing PT goals (*p* = 0.013, Exp (B) = 0.209), while WLGS ≥ 3 was no longer significant (*p* = 0.158, Exp (B) = 0.368). However, pelvic weakness significantly predicted a lower likelihood of goal completion (*p* = 0.033, Exp (B) = 0.236).

Sensitivity analysis confirmed that breast cancer status and olanzapine had no impact on SMI at last PT, or the number of PT goals completed (*p* ≥ 0.48), but that SMI at baseline assessment was predictive of final SMI (*p* = 0.04) and that the number of PT goals set was predictive of PT goal completion (*p* < 0.001).

## 4. Discussion

Recent studies have demonstrated that low-dose olanzapine significantly increases advanced cancer cachexia patient weight and appetite, while simultaneously reducing nausea [16]. This study aimed to investigate whether these benefits extended to improvements in muscle mass and functional capacity with targeted rehabilitation. However, in this selected population with appetite concerns, undergoing physical therapy for related impairments, olanzapine did not result in additional measurable benefit in patient outcomes beyond the multi-modal rehabilitation already offered in the rehabilitation clinic [30,31]. While subjective appetite and nausea were not collected at the conclusion of the rehabilitation period, OLZ+ body weight did not increase compared to baseline or to the OLZ- group. Weight stabilization was achieved in both groups, with the observed significant decrease in BW prior to first encounter not persisting through rehabilitation. Additionally, SMI and PNI remained stable, indicating that muscle mass and nutritional status were not additionally improved by olanzapine beyond the rehabilitation clinic standard care.

Importantly, this population was engaging in active rehabilitation and targeted exercise, which can modulate metabolism independently. Thus, precision dietary guidelines may need to accompany olanzapine in the context of cancer rehabilitation. Although the sample size was small, the groups were well matched at baseline. Sensitivity analysis also revealed that the difference in SMI between groups was not significantly impacted by covariates, including time elapsed between evaluation and PT initiation, or breast cancer diagnosis.

Reflecting this clinical similarity, there was no statistically significant difference in PT outcomes across groups. While representing the clinical, real-world nature of this study, PT goals are an exploratory outcome and may be subject to variability between physical therapists and goal types. The only notable difference was that OLZ- patients had similar MMT scores across lower extremity muscle groups, whereas OLZ+ patients demonstrated differential strength in hip flexion versus abduction and extension. This parallels our lab’s work, in Dong et al. (2025), identifying gluteal strength as an impairment specific to cachexia [30]. These findings may indicate that the OLZ+ group may have skewed towards functionally affected cachexia more so than the OLZ- cohort. Moving forward, this suggests that future studies should more comprehensively assess functional status, in addition to body composition, when assessing the impact of orexigenic medications.

In particular, the potential for unwanted interactions between medication side effects and disease-related impairments must be addressed in these multifactorial populations. Relevantly, a secondary finding of this study is that fatigue was a significant predictor of PT goal completion. Fatigue is the most prevalent symptom experienced by cancer patients [32]. This form of fatigue is more severe, is not due to physical exertion, and cannot be resolved with rest and sleep [33]. Unsurprisingly, fatigue is closely related to physical function in patients undergoing cancer-related rehabilitation [34]. This relationship is particularly vital when discussing olanzapine as a treatment for cancer-related weight loss, as fatigue is one of the common side effects [35,36].

While olanzapine may be effectively addressing the negative effects of nausea and anorexia, it may not be sufficient to mitigate the prevalent cancer-related fatigue. As fatigue and drowsiness are side effects of olanzapine, it is possible that baseline impairments of fatigue were augmented in the OLZ+ group. More likely, pre-existing cancer fatigue serves as an independent co-factor in functional trajectory that cannot be mitigated by olanzapine. This further underscores the importance of holistic, multidisciplinary care when treating a highly multifactorial disease. Appetite stimulation alone is insufficient to restore function if underlying issues, such as fatigue, remain unaddressed.

There are a few limitations to consider when discussing these outcomes. The study was retrospective, creating high possibility of selection bias. For example, if a patient did not report distress with appetite, they would not be flagged in the chart review process. There was no quantitative measurement of food intake, such as the Food Frequency Questionnaire or a dietary recall, and there was no way to measure patient adherence to olanzapine. Additionally, the observational nature of the study cannot account for covariates that influence patient decision making about taking olanzapine or not, and as a result does not allow for the inference of any causal effects. Social support, financial burden, and psychological status are all factors that influence rehabilitation success, but could not be measured in this study [37].

## 5. Conclusions

Olanzapine is a useful tool for regulating nausea and appetite in a cancer palliative setting, yet it remains inconclusive whether those effects extend beyond the physical interventions that are provided to cancer patients in the rehabilitation setting. The role of olanzapine in improving functional outcomes during active cancer rehabilitation requires further investigation into adjuvant therapies that specifically target fatigue and muscle preservation.

## Figures and Tables

**Table 1:** Sample Characteristics. Demographic and clinical characteristics of patients receiving olanzapine (OLZ+) compared to those not receiving olanzapine (OLZ−). Data are presented as Mean (SD) for continuous variables, or Count [%] for categorical variables. Statistical differences between treatment groups were assessed using an independent samples *t*-test for continuous variables and Fisher’s exact tests for categorical variables. No significant differences were observed between groups across any variables, except for breast cancer prevalence (*p* = 0.027).

Characteristics	Olanzapine Patients Mean (SD) or Count [%]	Non-Olanzapine Patients Mean (SD) or Count [%]	*p* Value
Sample Population			
Age at evaluation OLZ+ (*n* = 20) vs. OLZ− (*n* = 25)	59 (18)	64 (12)	0.277
Male OLZ+ (*n* = 20) vs. OLZ− (*n* = 25)	9 [45]	11 [44]	1.00
Days to PT evaluation from 1st reported appetite issue OLZ+ (*n* = 16) vs. OLZ− (*n* = 25)	55 (83)	47.9 (95)	0.803
Days spent completing PT OLZ+ (*n* = 19) vs. OLZ− (*n* = 23)	76 (55)	63 (46)	0.417
Cancer Types			
Pancreatic	6 [67]	3 [12]	0.157
Upper GI	0 [0]	3 [12]	0.242
Lower GI	5 [25]	3 [12]	0.435
Lung	5 [25]	3 [12]	0.435
Breast	0 [0]	6 [24]	**0.027**
Gynaecologic	0 [0]	2 [8]	0.495
Hematologic	4 [17]	1 [4]	0.155
Endocrine	0 [0]	4 [16]	0.117
Other	2 [10]	2 [8]	0.815
Cancer Stage			
I	1 [7]	2 [9]	0.837
II	0 [0]	3 [14]	0.149
III	3 [21]	7 [32]	0.497
IV	10 [74]	10 [45]	0.126
Fearon +OLZ+ (*n* = 19) vs. OLZ− (*n* = 23)	12 [60]	13 [52]	0.540
WLGS ≥ 3OLZ+ (*n* = 17) vs. OLZ− (*n* = 24)	12 [60]	13 [52]	0.344
Impairments			
Not independent with ADLs OLZ+ (*n* = 12) vs. OLZ− (*n* = 18)	1 [5]	3 [12]	0.622
Not Independent with IADLs OLZ+ (*n* = 12) vs. OLZ− (*n* = 14)	5 [25]	10 [40]	0.238
Neuropathy OLZ+ (*n* = 20) vs. OLZ− (*n* = 25)	5 [25]	6 [24]	1.00
Ataxia OLZ+ (*n* = 20) vs. OLZ− (*n* = 25)	9 [45]	6 [24]	0.205
Fatigue OLZ+ (*n* = 19) vs. OLZ− (*n* = 25)	15 [75]	17 [68]	0.745
Pelvic/Glute Weakness OLZ+ (*n* = 19) vs. OLZ− (*n* = 25)	16 [72]	18 [80]	0.745

Bolding of in the *p*-value column denotes significance.

**Table 2:** Comparing demographics and impairments at evaluation to the proportion of PT goals complete, via beta binomial model. Analysis of the number of physical therapy (PT) goals assigned and completed between patients receiving olanzapine (OLZ+) and those not receiving olanzapine (OLZ-). Exp B indicates the adjusted odds ratio. 95% CI refers to the 95% Wald confidence interval for Exp B; p represents the significance level calculated via Wald chi-squared test (bolded where *p* < 0.05). Model standard errors were corrected using the corresponding Pearson chi-squared dispersion scaling factor.

Source	Exp B	95% CI	*p*
Age	1.022	[0.986–1.059]	0.243
OLZ+ (*n* = 20) vs. OLZ− (*n* = 25)	0.802	[0.285–2.257]	0.675
Male	0.844	[0.301–2.369]	0.748
OLZ+ (*n* = 20) vs. OLZ− (*n* = 25)	0.861	[0.307–2.419]	0.777
Fatigue	0.271	[0.092–0.793]	**0.017**
OLZ+ (*n* = 19) vs. OLZ− (*n* = 25)	0.789	[0.276–2.256]	0.658
Pelvic Weakness	0.507	[0.172–1.496]	0.219
OLZ+ (*n* = 19) vs. OLZ− (*n* = 25)	0.848	[0.303–2.371]	0.753
SMI	0.646	[0.382–1.092]	0.103
OLZ+ (*n* = 15) vs. OLZ− (*n* = 16)	0.364	[0.088–1.365]	0.130
Neuropathy	2.078	[0.673–6.416]	0.204
OLZ+ (*n* = 15) vs. OLZ− (*n* = 16)	0.838	[0.299–2.350]	0.737
Ataxia	1.108	[0.363–3.387]	0.857
OLZ+ (*n* = 15) vs. OLZ− (*n* = 16)	0.887	[0.312–3.387]	0.882
Independent with ADLs	1.460	[0.203–10.500]	0.707
OLZ+ (*n* = 15) vs. OLZ− (*n* = 16)	0.691	[0.183–2.612]	0.586
Fearon Criteria Positive	0.368	[0.117–1.151]	0.597
OLZ+ (*n* = 19) vs. OLZ− (*n* = 23)	0.734	[0.233–2.312	0.086
WLGS ≥ 3	0.255	[0.079–0.819]	**0.022**
OLZ+ (*n* = 17) vs. OLZ− (*n* = 24)	0.556	[0.172–1.805]	0.329

## Data Availability

Data will be made available upon reasonable request.

## Abbreviations

The following abbreviations are used in this manuscript:

PT	Physical Therapy
ASCO	American Society of Clinical Oncology
OLZ+	Patients taking olanzapine
OLZ-	Patients not taking olanzapine
BMI	Body Mass Index
GI	Gastrointestinal
WLGS	Weight Loss Grading Scale
MMT	Manual Muscle Testing
BW	Body Weight
